# Investigation of DNA variants specific to *ROBO2* Isoform ‘a’ in Irish vesicoureteric reflux patients reveals marked CpG island variation

**DOI:** 10.1038/s41598-020-58818-6

**Published:** 2020-02-10

**Authors:** John M. Darlow, Mark G. Dobson, Andrew J. Green, Prem Puri, David E. Barton

**Affiliations:** 1 Department of Clinical Genetics, Dublin, Ireland; 2 National Children’s Research Centre, Dublin, Ireland; 3 School of Medicine and Medical Science, University College Dublin – all at Our Lady’s Children’s Hospital, Crumlin, (now renamed ‘Children’s Health Ireland at Crumlin’), Crumlin, Dublin, D12 N512 Ireland; 4 Beacon Hospital, Beacon Court, Sandyford, Dublin, D18 AK68 Ireland

**Keywords:** Development, Gene expression, Mutation, Genetics research, Paediatric research

## Abstract

*ROBO2* gene disruption causes vesicoureteric reflux (VUR) amongst other congenital anomalies. Several VUR patient cohorts have been screened for variants in the ubiquitously expressed transcript, *ROBO2b*, but, apart from low levels in a few adult tissues, *ROBO2a* expression is confined to the embryo, and might be more relevant to VUR, a developmental disorder. *ROBO2a* has an alternative promoter and two alternative exons which replace the first exon of *ROBO2b*. We screened probands from 251 Irish VUR families for DNA variants in these. The CpG island of *ROBO2a*, which includes the non-coding first exon, was found to contain a run of six variants abolishing/creating CpG dinucleotides, including a novel variant, present in the VUR cases in one family, that was not present in 592 healthy Irish controls. In three of these positions, the CpG was created by the non-reference allele, and the reference allele was not the nucleotide that would result from spontaneous deamination of methylcytosine to thymine, suggesting that there might have been selection for variability in number of CpGs in this island. This is in marked contrast to the CpG island at the start of *ROBO2b*, which only contained a single variant that abolishes a CpG.

## Introduction

*ROBO2* is one of at least twenty genes implicated in non-syndromic vesicoureteric reflux (VUR), though the majority of genetic aetiology of VUR is still unknown. VUR is the reverse flow of urine from the bladder towards the kidneys, and results from a developmental defect in the angle and position of insertion of the ureter through the bladder wall. The ROBO2 protein is the receptor for SLIT2. ROBO/SLIT signalling is involved in axon guidance and neuronal migration but the proteins are expressed in many tissues and may have functions during other cell migration processes, many of which are yet to be elucidated. The critical first step of development of the metanephric urinary system is the emergence of the ureteric bud from a caudal location on the mesonephric (Wolffian) duct. Slit2/Robo2 signalling has been shown to be one of the mechanisms regulating the precise position of the emergence of the ureteric bud, and in the mouse it was shown that inactivation of both copies of either *Slit2* or *Robo2* leads to the emergence of multiple buds^1^. Disruption of a single copy of *ROBO2* in a child with a 3;Y translocation through the gene (on Chromosome 3) was found to be the cause of multiple congenital abnormalities, including severe bilateral VUR with ureterovesical junction defects^2^. Screening of patients with non-syndromic VUR has revealed a number of non-synonymous variants in *ROBO2* not found in controls^2–6^, though other such variants, predicted to be damaging, have been found in controls^4^, and yet other such variants have been found in other non-syndromic congenital anomalies of the kidneys and urinary tract^6^, and another was found in a fetus with bilateral renal and bladder agenesis and a hypertrophic heart, which also had a non-synonymous *SLIT2* variant^7^.

**Figure 1:**
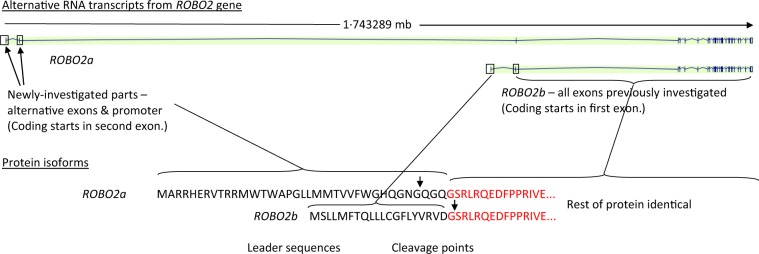
*ROBO2a* RNA transcripts and protein sequences. Exon 2 of *ROBO2a* codes for the 36 amino-acids of the ROBO2a leader sequence. Exon 1 of *ROBO2b* codes for the 20 amino-acids of the ROBO2b leader sequence. The rest of the protein is coded by the other 25 exons from Exon 3 of *ROBO2a*, which is Exon 2 of *ROBO2b*. After cleavage of the leader sequences, the mature ROBO2a protein has just 4 more amino-acids than ROBO2b. Diagram modified from Yue *et al*.^8^; the transcript displays in our figure are from the University of California Santa Cruz (UCSC) Genome Browser.

Having screened our own VUR cohort for such variants in the 26 exons of the ubiquitously-expressed isoform, now known as *ROBO2b*^4^, which was the only transcript shown in the genome browsers when the first screen of VUR patients was carried out^2^, we decided that we should check the alternative promoter and alternative exons of another transcript, named *ROBO2a* in harmony with the naming of previously-discovered isoforms of *ROBO1*, *ROBO1a* and *ROBO1b*^8^. The first two exons of *ROBO2a* are more than 1 mb to 5′ of the 26 exons of *ROBO2b*, and the second exon of *ROBO2a* is spliced to the second exon of *ROBO2b*, making a transcript of 27 exons. The encoded protein, the ROBO2a precursor, has 36 amino-acids at its N-terminus not present in the precursor ROBO2b, and the ROBO2b precursor has 20 amino-acids not present in the ROBO2a precursor, but after cleavage (at slightly different positions) of the signal peptides, the mature ROBO2a protein just has four extra N-terminal amino acids compared with ROBO2b and the rest of the protein is identical (Fig. 1). Many proteins have on their N-termini a series of amino-acids known, as a signal peptide or leader sequence, that direct the protein through a particular membrane, such as the rough endoplasmic reticulum or the mitochondrial membrane. These peptides are cleaved from the rest of the protein by proteolytic enzymes after they have passed through the membrane. The different leader sequences of ROBO2a and ROBO2b, suggest the possibility that they direct the mature receptor protein to different physical locations, but this is as yet undetermined. Yue *et al*.^8^ showed that though ROBO2b is expressed in most adult tissues, ROBO2a is only expressed at low levels in a few adult tissues. They found ROBO2a to be highly expressed in human fetal brain, but did not examine fetal urinary tract tissue. However, in the mouse, they examined expression of the isoforms in whole embryo extracts from 11·5, 13·5 and 16·5 days post conception (dpc), as well as various adult tissues, and found the greatest expression of Robo2a at the earliest embryonic stage, gently declining with gestation, and no expression in the adult tissues except the brain. GUDMAP, the GenitoUrinary Development Molecular Anatomy Project database^9,10^, shows expression of Robo2 in the metanephric mesenchyme at 11·5 dpc in the mouse embryo (Cathy Mendelsohn Lab microarray data), but does not distinguish between the isoforms. Our decision to check the alternative exons and alternative promoter of ROBO2a was not just for completeness, but because of the possibility that ROBO2a might be the more relevant isoform in embryonic development.

In the meantime, rebuilding and annotation of the Human Genome has continued, and the picture is now highly complex, and clearly far from being resolved. Six more exons have been recognised, bringing the total up to 34. One of these is located, in the long gap between Exon 2 of *ROBO2a* and Exon 1 of *ROBO2b*. The other five - of 12, 139, 126, 183 and 273 bp in length, lie between the exons that are numbers 6 & 7, 11 & 12, 20 & 21, 23 & 24, and 24 & 25 of *ROBO2b* respectively, but they are all in other transcripts (most of them coding for longer proteins as a result) and not present in *ROBO2a* or *ROBO2b*. The three genome browsers all differ in numbers and details of *ROBO2* transcripts and have changed their displays several times during the work reported here. It seems that there may be over 30 different coding transcripts, as well as several non-coding ones, and possibly as many as ten different starting sites. It may be some time before all of the transcripts and protein products are validated, and longer before the spatial and temporal expression of each is mapped in the adult, let alone in individual cell types in human embryos (if expressed then). However, *ROBO2a* expression is known to be almost restricted to the embryo. Variation of its specific starting exons and upstream sequence have not previously been reported in VUR patients, and we find an interesting difference compared with the start of *ROBO2b* which may be of interest beyond the topic of VUR. This investigation was complicated by the presence of non-coding copies of the *ROBO2a* exons on Chromosomes 20 and 22.

## Results

### Initial screen for DNA variants

Index cases from our VUR families were screened for DNA variants in the promoter and exons specific to *ROBO2a* by commercial Sanger sequencing (plus in-house work where necessary – see Materials and Methods) and then followed up with in-house investigations including family and control studies. (Note that though we had DNA from parents of VUR cases, it was usually not known whether either of the parents had had VUR.) Exon 1 is entirely non-coding. Authorities currently disagree on the position of the start of the exon, making it 266, 267 or 286 bp in length (see Supplementary Information) but we can say that the sequenced region covering the promoter and Exon 1 was 1,543 bp finishing 102 bp downstream of the exon. There is a very similar copy of this exon and its surrounding sequence on Chromosome 22q11.1, but the four overlapping pairs of commercial primers successfully amplified only the Chromosome 3 sequence. Nine variants were found in the upstream and first-exon amplicons in this initial screen: the first nine detailed in Table 1. Four of these variants (two found in only one index case each and two in two index cases each) were not recorded in the version of dbSNP current at the time, so the samples from the families of the index cases who bore them were investigated for segregation of the variant alleles with VUR. (They are the first four variants for which family studies are shown in the family studies columns of Table 1, *i.e*. the variants at c.-1386_-1384, c.-320, c.-111 and c.-78, as listed in the Position column of Table 1.) We used in-house primers, in order that we could screen by high-resolution melting-curve (HRM) analysis, because the commercial sequencing amplicons are 500–600 bp in length and the optimum amplicon length for detection of sequence variants by HRM is 150–400 bp. The first of the novel variants (c.-1386_-1384del) was in a gap in the homology with Chromosome 22 (see Supplementary Fig. S1) so it was possible to design a primer-pair specific to Chromosome 3. However, the region immediately surrounding the other three novel variants (c.-320, c.-111 and c.-78) is very similar to the Chromosome 22 sequence, so nested PCR was performed using the Chromosome-3-specific commercial primer-pair in the first round followed by internal amplification of the PCR products with HRM primers (see Materials & Methods).

**Table 1:** DNA variants upstream and in the first two exons of *ROBO2a* and their flanking regions in VUR index cases.

Index case screen												Family studies			Control studies				
hg19 co-ord	hg38 co-ord	Region	Position	DNA change	Creates/removes CpG	HV	Het	HR	Allele freq	dbSNP No.	Cons.RS (GERP)	VUR + var	VUR − var	No. of fams	Allele freq	HV	Het	HR	Source
75,954,739–41	75,905,588–90	Upstream	c.-1386_-1384del	delATC TTATCAT → TTAT	No	0	1	237	0.0021	rs748917584	0.714	1	1	1	0.0005	0	7	14,994	gnomAD-NFE
75,954,807	75,905,656	Promoter	c.-1318	AGG → AAG	No	0	6	232	0.0126	rs150510983	0.452	—	—	—	0.0165	0	3	88	1000 GP-GBR
75,955,183	75,906,032	Promoter	c.-942	AGA → ATA	No	0	2	237	0.0042	rs151063300	−0.468	—	—	—	0.0055	0	1	90	1000 GP-GBR
75,955,760	75,906,609	Promoter	c.-365	***GCG → GTG***	***Removes***	78	113	60	0.5359	rs35862955	0.328	—	—	—	0.5330	25	47	19	1000 GP-GBR
75,955,805	75,906,654	Promoter	c.-320	***AGG → ACG***	***Creates***	0	2	249	0.0040	rs754253590	1.27	2	4	2	0.0068	0	8	584	This study
75,955,894	75,906,743	5′UTR	c.-231	**GAG → G*CG***	***Creates***	0	10	241	0.0199	rs34678735	2.49	—	—	—	0.0304	1	34	557	This study
75,956,014	75,906,863	5′UTR	c.-111	***CGC → CAC***	***Removes***	0	1	250	0.0020	—	1.56	2	0	1	0	0	0	592	This study
75,956,047	75,906,896	5′UTR	c.-78	**G*CG → GTG***	***Removes***	0	2	249	0.0040	rs532303838	1.55	2	3	2	0.0036	0	54	15,002	gnomAD-NFE
75,956,058	75,906,907	5′UTR	c.-67	***CAG → CCG***	***Creates***	13	91	147	0.2331	rs13073919	−4.74	—	—	—	0.2308	2	38	51	1000 GP-GBR
75,956,283	75,907,087	IVS1	c.-14 + 127	***GCT → GTT***	***No***	0	2	249	0.0040	rs182521620	0.303	3	0	2	0.0017	0	2	590	This study
75,986,568	75,937,417	IVS1	c.-13–64	TCG → TTG	Removes	0	17	234	0.0339	rs539728114	0.236	—	—	—	0.0330	0	6	85	1000 GP-GBR

‘Position’ is based on the ‘A’ of the initiation codon methionine (‘ATG’) at position 300 of NCBI Reference Sequence NM_001128929.3 (GI: 586946395) = nucleotide 1. However, this reference sequence begins at c.-299, and positions 5′ to that can be found on the genomic reference sequence NC_000003.12 (GRCh38.p7 Primary Assembly) using any genome browser and the hg38 co-ordinates given in column 2 above. Description of the variants is as recommended by HGVS (www.hgvs.org/mutnomen). ‘DNA change’ means the change relative to the allele currently held to be the reference allele. The first variant listed is a deletion of 3 nucleotides. In the gnomAD database, this variant is described as residing at one place to 5′ of the HGVS starting position, i.e. at 75,954,738 hg19, and having alleles as Reference ‘TATC’ and Variant ‘T’, whereas we have used the HGVS recommended description, delATC. To illustrate the change, we have shown the two reference nucleotides on either side of the 3 deleted. All the other variants listed are single nucleotide substitutions, but we show one nucleotide on either side of the variant position. The reference sequence is on the left; the variant sequence is on the right. ‘Creates/removes CpG’ also applies relative to the reference allele, and the CpG removed or created by the variant allele is underlined. The bold and italics indicates the variants within the 549-bp *ROBO2a* CpG island (see main text). Abbreviations: HV, homozygous variant; Het, heterozygote; HR, homozygous RefSeq allele; Cons., conservation in mammals by ‘rejected substitution’ (RS) score by Genomic Evolutionary Rate Profiling (GERP); VUR + var, number of VUR cases found to have the variant; VUR – var, number of VUR cases not having the variant in the same families; gnomAD-NFE allele and genotype frequencies in non-Finnish Europeans in the gnomAD database; 1000 GP-GBR, allele and genotype frequencies in the ‘British in England and Scotland (GBR)’ subset of the 1000 Genome Project data.

These investigations showed that three of the four novel variants did not segregate with VUR - at least one family with each contained a member who had VUR but did not carry the variant (as shown in the ‘VUR – var’ column of Table 1) – and all of these variants have subsequently appeared in more recent versions of dbSNP. The other variant, at position -111 in the 5′-UTR, was found to be carried by both affected children and the father in the one family in which it occurred, Family 135. We therefore screened our 592 Irish population control samples for this variant, and it was not present, making it a potential candidate for causing VUR. Exon 2 is 122 bp in length, the first 13 bp non-coding before the translation start codon, so can be covered with generous flanking sequence by a single amplicon. However, the complication was again genomic copies, with homology extending far beyond the exon on either side. Yue *et al*.^8^ had warned that there were copies of Exons 1 & 2 on 20p11.1 and 22q11.1, and we had found that the Exon 1 sequence had a match on Chromosome 22, so were expecting a Chromosome 20 match for Exon 2. The results of the commercial sequencing of Exon 2 in our VUR index cases suggested that at least two different genomic copies had been amplified, as 18 variants were reported in a 535-bp amplicon, and our inspection of the chromatograms showed other positions in which there were also two different nucleotides, which had missed detection by the sequence-analysis software. We therefore submitted the Exon 2 sequence to GRCh37/hg19 build of the Human Genome by BLAT, and this indicated a single complete copy on Chromosome 20 and showed that the reverse commercial primer matched both copies perfectly, and the forward one differed from the Chromosome 20 sequence only at one position, 12 nucleotides back from the 3′ nucleotide of the primer. Many of the variants corresponded with reported SNPs, so we designed Chromosome 3- and Chromosome 20-specific primers so that we could determine which were true SNPs on Chromosome 3, which were true SNPs on Chromosome 20, and which were not SNPs at all but merely differences between the fixed sequences at the two genomic locations, in addition to identifying any novel variants. PCRs with the new primers revealed that there must be more genomic copies. The GRCh38/hg38 build of the genome showed two more copies, both thought to be on Chromosome 22. We eventually accounted for all variations in the sequences that we obtained. Full details are given in the Supplementary Information and Supplementary Table S1, but the salient point is that there was found to be only one variant in the Chromosome 3 copy, the true *ROBO2a* Exon 2 amplicon. It is a polymorphism, now known as rs539728114, and is not in the exon but 64 bp upstream of it (this is the last variant listed in Table 1). Thus the initial screen of the two exons, their flanking regions and sequence upstream of Exon 1 had found ten variants of which one was a possible cause of VUR. However, enquiry into variant function showed that more investigations were warranted.

### Regulatory context of the novel variant at c.-111 and further investigations

The novel variant is in the 5′-UTR of the gene. The variant allele is an adenosine which replaces the guanosine of a CpG dinucleotide, thereby removing this CpG site. The cytosine of this CpG dinucleotide was found to be methylated on 100% of DNA molecules in 5/23 cell lines in which methylation was investigated by bisulphite sequencing in the ENCODE project, and methylated on 50% of the DNA molecules in a further two cell lines, and the genome there was found to have the histone methylation H3K4Me3 mark, often found near promoters and the H3K27Ac mark often found near active regulatory regions (all annotated on the GRCh37/hg19 build of the Human Genome in the UCSC Genome Browser). This suggested the possibility that this variant might alter the expression of *ROBO2a* in some cell types, though unfortunately few cells of urinary tract origin were included in the ENCODE project and none were tested for methylation.

**Figure 2:**
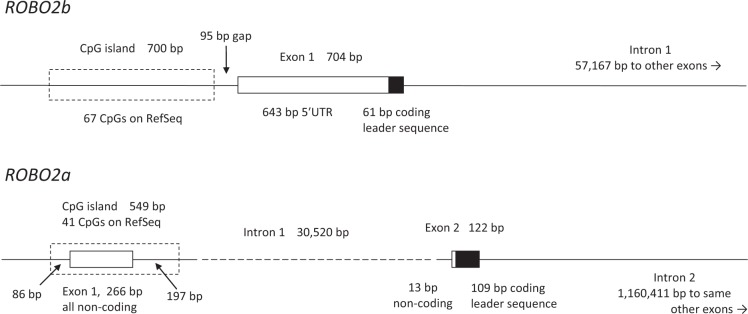
Comparison of the arrangement of CpG island, 5′ untranslated region (□) and coding (■) of the protein leader sequence in the *ROBO2a* isoform with the arrangement of the same elements of the *ROBO2b* isoform.

A check of the other nine variants revealed that all but the three furthest from the start of the gene either created or removed a CpG with respect to the reference allele. For each, one allele made a CpG and the other did not, and the one that made the CpG was not always the reference allele and was not always the major allele. Seven in a row (counting the novel variant) seemed to require an explanation. The last variant of these seven is over 30 kb from the rest, 64 bp upstream of Exon 2, but the first six were observed to be in a CpG island, chr3:75,955,760–75,956,308 hg19, chr3:75,906,609–75,907,157 hg38. The arrangement of the start of *ROBO2a* is different from that of *ROBO2b* (Fig. 2). *ROBO2b* has a CpG island which is entirely upstream of the first exon, in which coding begins after a long 5′UTR, but the CpG island at the start of *ROBO2a* entirely surrounds the first exon, which is all non-coding, and extends further downstream than the flanking region that we had screened.

We therefore designed a new set of Chromosome 3-specific primers (to avoid amplifying the Chromosome 22 copy) and screened the remaining 99 bp of the island by PCR and HRM analysis followed by sequencing of cases with aberrant melting curves. This uncovered a single variant, present in two index cases. It is rs182521620 and does not affect a CpG dinucleotide. (This is the penultimate variant shown in Table 1.) dbSNP has no frequency data for it, and the Genome Aggregation Database (gnomAD) gives a minor-allele frequency of 9/15,006 in non-Finnish Europeans (*i.e*. 9 heterozygotes out of 7,503 individuals), giving it a frequency of 0·0005998, which is much rarer that 2/251 index cases, so first we checked the families for segregation with VUR. Unfortunately this was not very useful due to lack of DNA from many family members of these two cases. For one of the index cases we only had DNA from the mother, who had the variant, and from the other family we had DNA from both parents and the affected sibling, and the sibling and the mother also had the variant. We could thus not exclude the variant from segregation with VUR, so screened our 592 healthy control samples. Two of them were heterozygotes for the variant, so it is evidently more common in Ireland than in other non-Finnish Europeans, and not significantly different from the frequency in our Irish VUR index cases. Our screen for variants in the start of *ROBO2a* had thus found a total of 11 variants (Table 1) and we still had a run of six CpG variants.

In contrast, a check of our results of *ROBO2b*, of which we had previously screened the upstream sequence and all exons in our index cases^4^, showed that we had also found eleven variant positions in the equivalent part of that isoform, but only two made a CpG for one allele and not for the other. They are adjacent variants but 451 bp apart and only one of them in the CpG island. The *ROBO2b* upstream CpG island, chr3:77,088,499–77,089,198 hg 19, is 700 bp long, and contains 67 CpGs in the Reference Sequence. However, only three of the variants in our index cases were in this island, and only the one caused the presence or absence of a CpG, while another merely moved a CpG (the alleles being Cp**C**pG and Cp**G**pG) and the third did not involve a CpG at all (Table 2). Genes may also have CpG islands within the gene body (defined as between 500 bp downstream of the transcription start site to the end of the last exon). Despite reportedly having many different transcripts with several different start sites, the whole human *ROBO2* gene has just three CpG islands: the ones at the beginnings of the *ROBO2a* and *ROBO2b* transcripts and a gene-body island that surrounds Exon 2 of *ROBO2b*. The latter island, chr3:77,147,168–77,147,399, hg19, chr3:77098017–77098248, hg38, is 232 bp long and contains 20 CpG dinucleotides. We found a single variant within this, rs6788280, which was also found by other investigators of *ROBO2b* in VUR patients, and that disrupts a CpG dinucleotide.

**Table 2:** DNA variants previously reported in our VUR index cases in the equivalent region of *ROBO2b* to the variants reported in *ROBO2a* in Table 1.

hg19 co-ord	hg38 co-ord	Region	Position	DNA Change	Changes CpG	dbSNP No.
77,088,313	77,039,162	Promoter	c.-1624	GCT → GTT	No	—
77,088,402	77,039,251	Promoter	c.-1535	CCA → CGA	Creates	rs73110408
77,088,753	77,039,602	Promoter	c.-1084	***TCG*** ***→ TGG***	***Removes***	rs11919722
77,088,759	77,039,608	Promoter	c.-1078	***GCC → GTC***	***No***	rs77461867
77,089,095	77,039,944	Promoter	c.-842	***CCG*** ***→*** ***CGG***	***Moves left M***	rs9835590
77,089,241	77,040,090	Promoter	c.-696	TCC → TTC	No	—
77,089,395	77,040,244	5′UTR	c.-592	TTC → TCC	No	rs3923745*
77,089,478	77,040,327	5′UTR	c.-459	GGG → GAG^‡^	No	—
77,089,698	77,040,547	5′UTR	c.-239	AGT → AAT	No	—
77,089,699	77,040,548	5′UTR	c.-238	GTG → GGG	No	rs3923744
77,090,112	77,040,961	IVS1	c.61 G + 115	TCC → TTC	No	rs114060047

All variants recorded in this table are single-nucleotide substitutions, but one nucleotide on either side of the variant position is also shown, as in Table 1, in order to show whether a CpG dinucleotide is present or not.

*This variant was not recorded in dbSNP at the time of publication of our ROBO2b paper (ref. ^4^). The variants without rs numbers, which were found in single index cases and are reported in Appendix 2 of ref. ^4^, are still not in dbSNP and none is present in gnomAD either.

^‡^This variant was erroneously reported in ref. ^4^ as having been found in only one PCR. On checking chromatograms for this paper, we find that it was present in two overlapping amplicons using different primer-pairs, so is unequivocal.

‘Position’ is based on the ‘A’ of the initiation codon methionine (‘ATG’) at position 644 of NCBI Reference Sequence NM_002942.4 (GI: 299116179) = nucleotide 1. However, this reference sequence begins at c.-643, and positions 5′ to that can be found on the genomic reference sequence NC_000003.12 2 (GRCh38.p7 Primary Assembly) using any genome browser and the hg38 co-ordinates given in column 2 above. The bold and italics indicates the variants within the 700-bp *ROBO2b* CpG island (see main text). ‘Moves left M’ means that the base-change shifts a CpG by one position to 5′ (it changes CpCpG to CpGpG) and that the cytosine of the reference CpG is methylated in some cell-lines of ENCODE. For other notes and abbreviations, see legend to Table 1.

**Figure 3:**
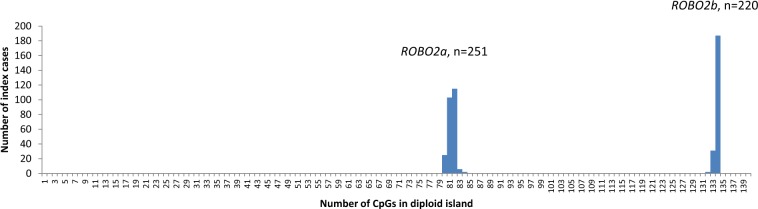
Variation in numbers of CpG dinucleotides in the CpG islands at the beginnings of the *ROBO2* ‘a’ and ‘b’ transcripts.

The run of variant CpG sites in the *ROBO2a* CpG island made it likely that nearly all individuals would be heterozygous for some of them, raising the possibility that this island might never be as highly methylated as the island at the start of *ROBO2b*. We therefore checked the genotypes of all six variants in all 251 VUR index cases (rather than the 592 controls, because most of the index case samples had been sequenced whereas the control samples had only been screened by high-resolution melting-curve analysis for the novel variant and the last variant discovered, and there was no reason to suppose that the VUR samples would be more variable than the controls, except for the presence of the novel variant). The full set of 6 × 251 genotypes is given in Supplementary Table S2. In the diploid sequence of the six variant CpG positions, the homozygous Reference Sequence would have six CpGs and the total possible number is 12. Our 251 cases had a mean of 5·430 CpGs, standard deviation 0·736, with a range of 4–8 CpGs. The phasing is unknown, so we cannot list the haplotypes, but we can say from the diploid genotypes that there were two different diplotypes with 4 CpGs, 3 with 5 CpGs, 4 with 6 CpGs, 2 with 7 CpGs and only one with 8 CpGs at the variable positions. The total number of all CpGs in the CpG island at the start of *ROBO2a* is less than in that of *ROBO2b*, and is of course more variable, since the latter has only one presence/absence CpG variant (Fig. 3).

Finally, we investigated the haplotype of the novel CpG-altering variant. We genotyped all six positions in both parents and the affected sibling of the index case with the variant, enabling us to derive the haplotypes. This revealed that both parents had diplotypes containing 5 CpGs, each having one haplotype with 3 CpGs and one with two. The haplotype with 3 CpGs was the same for both parents and was the reference sequence haplotype, while the haplotypes with two CpGs were different. Both children had diplotypes containing only 4 CpGs, having both received the haplotypes with 2 CpGs from both parents. The one from the mother had the non reference allele ‘T’ of rs35862955 at c.-365 removing one CpG from the reference haplotype. The one from the father also had the non-reference allele of rs35862955 removing one CpG, and had the novel allele at c.-111 removing another CpG, but gained a CpG from having the non-reference allele of rs13073919 at c.-67. The genotypes and haplotype derivations of all four individuals are also shown in Supplementary Table S2.

## Discussion

This is the first report of investigation of the alternative exons and alternative promoter specific to the *ROBO2a* isoform in a group of patients. We decided to examine them in our VUR patients because Robo2a is expressed mainly in the embryo, and vesicoureteric reflux is caused by a developmental anomaly in a process occurring early in embryonic life. This process is the insertion of the ureter into the urinary bladder, but it has been established^11^ that the position of insertion is intimately related to the position at which the ureteric bud emerges from the mesonephric (Wolffian) duct (subsequently borne out by much work on mice) and *ROBO2* is one of the genes involved in the regulation of this emergence. The prevalence of VUR is unknown, because currently screening would require invasive investigation, but it is probably about 3% at birth. However, since many genes are known to be involved in the early development of the urinary tract with the potential to cause VUR, and because the expression of any gene may be affected by many different DNA variants, one would expect that any individual variant causing VUR will be rare. We did not, therefore, expect to find many variants with the potential to cause VUR in this study, but the promoter and exons specific to the *ROBO2a* isoform nonetheless seemed a worthwhile site in which to look.

In the index cases from 251 families, we found eleven variants, all non-coding, ten of which are now recognized as polymorphisms, but the other is unknown from any other investigation, was present in both affected children in one of our families, and was not present in our 592 healthy controls. We have found that this variant adds to an unexpectedly high proportion of variants around the beginning of the *ROBO2a* DNA sequence that affect the presence or absence of CpG dinucleotides, in particular adding one more to a run of five such variants in the CpG island at the start of the gene, and that this preponderance is in marked contrast to our findings around the beginning of the *ROBO2b* isoform.

Regions of high density of CpG dinucleotides, known as CpG islands, occur around the start sites of many genes, where they act as regulators, because methylation of the cytosines causes silencing of the gene. The CpG island at the start of *ROBO2a* is 549 bp long and contains 41 CpG dinucleotides in the reference sequence. Thus, substitution of just under 15% of the bases would abolish a CpG, and even supposing that an equal number of base-substitutions would create a new CpG, that would still only be a 30% chance that any random substitution within the island would affect a CpG, and 0·3^6^ = 0·000729, so six variants in a row all affecting CpGs would be unlikely by chance.

CpG dinucleotides tend to be methylated, a condition that helps to distinguish between the template strand and the new strand during proof-reading when the DNA has been replicated in cell-division. Because of this, CpG dinucleotides are less common than expected throughout most of the genome because methylated cytosines tend to convert to thymine by spontaneous deamination, and therefore CpGs tend to disappear during evolutionary time except where there are processes that maintain them, as in the regulatory islands at the start of genes^12^. This might suggest that our finding of six variants in a row that abolish or create CpG dinucleotides might not be unusual because loss of CpG dinucleotides by C-to-T mutation might be more common than mutation of any other nucleotides. However, as can be seen from Table 1, this cannot be the explanation. The alleles expected (stating the reference allele first) are C/T and G/A (if the cytosine mutated was on the reverse strand). Of the six variant positions, three abolish CpG dinucleotides relative to the Reference Sequence and three make new CpG dinucleotides relative to the Reference Sequence. The latter category could be instances in which the derived allele (T or A) just happened to be the one on the Reference Sequence, but they are not. One of these variant positions has alleles G/C and the other two A/C. This suggests the possibility that in the CpG island at the start of *ROBO2a* there has been selection for variability of the number of CpGs. This may be relevant to a difference in expression between the ‘a’ and ‘b’ isoforms of *ROBO2*.

In a study of methylation changes during induction of human fibroblasts to pluripotent stem cells, Deng *et al*.^13^ examined methylation of 2,020 CpG islands across two human chromosomes in 12 cell lines including fibroblasts, embryonic stem cells and induced pluripotent stem cells (IPSCs). They divided the CpG islands into those at the beginnings of genes, those in gene bodies and intergenic islands. Across all cell-lines they found that, of the islands at the beginnings of genes, 4·8% showed intermediate methylation, between 20% and 80%, and in a subsequent paper^14^, they calculated from the same data that in IPSCs, the proportion of these CpG islands with intermediate methylation, redefined as 25–75% methylated, was 7·6% (*i.e*. they found a higher percentage in these cells despite using a narrower range). In the latter paper, they examined the nature of this intermediate methylation and found that it was due to allele-specific methylation. While some SNPs within CpG dinucleotides determined only the methylation at the single site, the alternative alleles of others could determine the methylation of numerous CpG sites on either side, in ranges of up to 900 bp, and this was often cell-type specific. Most of the SNPs having this effect were in CpG dinucleotides, but some did not make a CpG with either allele. The authors concluded that the SNPs acted through affecting methylation regulators. Others have confirmed that single-nucleotide variants in CpG dinucleotides can affect the methylation of nearby CpG sites and are frequently quantitative trait loci^15^. Furthermore, as we submit this, a paper examining methylation in twins^16^, has found that the average contribution of additive genetic influences on DNA methylation across the genome is relatively low, but it is notably elevated at highly variable sites characterized by intermediate levels of methylation.

The emergence of the ureteric bud from the mesonephric duct is controlled by numerous stimulatory and inhibitory mechanisms that normally ensure that a single bud emerges in the optimum position. SLIT2/ROBO2 signalling is one of the inhibitory signals, and knock-out or reduction of *Robo2* expression can cause multiple budding (resulting in duplex urinary systems) and VUR^1,2^, whereas increased *Robo2* expression causes reduction in ureteric bud branching and reduction of numbers of nephrons^17^. As our novel variant removes a CpG dinucleotide, one might expect that it could reduce overall methylation of the CpG island and so increase expression of *ROBO2a* during embryonic development and would therefore be likely to cause renal dysplasia but not VUR. However, it has been shown that some SNPs within CpG sites can act, in some cell-types, in the opposite direction to that expected, with the allele removing a CpG resulting in increased methylation of the surrounding CpGs^14^. While, of course, we do not know whether the variant is a cause of VUR, we hope that others will also investigate the start of Isoform ‘a’ in their patient sets, and will be interested to know whether this reveals further variable CpG sites. Many gene expression studies have included *ROBO2* but we have found none that examine expression of individual transcripts. A very few studies have examined expression of *ROBO2* in relation to methylation (*e.g*. treatment of head & neck tumour samples with a demethylating agent caused increased *ROBO2* expression^18^) but again none investigated separate isoforms. Our investigation was a gene variant search, not an expression study, but we believe that it is the first to suggest that the different isoforms of ROBO2 might be regulated differently by methylation.

Comparison of genome builds reveals that for some SNPs, the alternative allele in the hg19 build has become the reference allele in the hg38 build and *vice versa*. This does not affect any of the SNPs that we report (though elsewhere it would affect some statements about whether a variant removes or creates a CpG relative to the reference sequence). However, it has resulted in the CpG island of *ROBO2b* being annotated as containing only 63 CpGs in the hg38 build, whereas the count was 67 in hg19, though the CpG island of *ROBO2a* has 41 in both builds.

## Materials and Methods

### Patients and families

The samples for this study were collected at Our Lady’s Children’s Hospital Crumlin, and the National Children’s Hospital, Tallaght, both in Dublin, Ireland. Ethical approval was granted by the research ethics committees of both hospitals, and informed consent was obtained from all subjects and/or their parents. Families with 2 or more affected members with primary VUR of any grade were collected. All families are Caucasian and the majority considered to be of homogeneous Irish ancestry. In 79 of these families one or more members has some other urinary or genital tract anomaly, of which duplex kidneys are by far the most common (50 cases from 48 families). Most index cases were referred because of recurrent urinary tract infections and all were diagnosed by micturating cysto-urethrograms (MCUGs). Sibs of index cases were screened by MCUG.

### Irish population controls

A DNA sample collection from peripheral blood samples from healthy members of the Irish population was established as the Irish Blood Transfusion Service – Trinity College Dublin (IBTS-TCD) BioBank, and we were given aliquots of 592 of these samples (296 male & 296 female).

### DNA variant detection and assessment

The first two exons, their intronic flanking regions and 1,176 bp upstream of the Ensembl-approved start of Exon 1 of *ROBO2a* were sequenced in whole-genome amplified DNA (Qiagen REPLI-g Services, Hilden, Germany) from 245 VUR index cases by Beckman Coulter Genomics (Beverley, MA, USA). Chromatograms reported to show variants were inspected visually to check for likely sequencing errors.

Variants that were not in dbSNP at the time, or were suspected of being artefacts due to duplicate sequences on other chromosomes, were then checked in our own laboratory in genomic DNA (not whole-genome amplified) with in-house primers by further PCRs and high-resolution melting curve (HRM) analysis, followed by Sanger sequencing of samples with deviant melting-curves. The sequences of all the primers, details of PCR mixtures, and cycling conditions are given in the Supplementary Information. For investigated variants upstream and in Exon 1, we checked the DNA from (a) six index case samples that had not been sent for sequencing (b) any of the 245 index cases whose samples had failed Beckman Coulter reporting for that variant and (c) members of families of any of the 251 index cases found to have the variant. Exon 1 has a copy on Chromosome 22, and where in-house primers were in positions in which the sequence on Chromosome 22 was identical or very similar to that on Chromosome 3, nested PCRs were performed within larger amplicons from primers in unique positions. To fill in the few missing genotypes of CpG variants in the CpG island in order to make a complete set of 6 × 251 genotypes (Supplementary Table S2) we amplified and sequenced with the ROBO2v1_04 primers (see Supplementary Information). For the variants that segregated with VUR, we screened Trinity BioBank control samples (see above) by the same method. For Exon 2, all index case samples were re-investigated using two different pairs of primers, specific to Chromosome 3 and to copies on Chromosomes 20 and 22, and variants were identified in sequences using Mutation Surveyor v4.05 (Softgenetics) and manual interpretation by comparison with the reference sequences.

Confirmed Chromosome 3 variants were assessed for conservation in mammals by Genomic Evolutionary Rate Profiling (GERP), previous reporting and predicted biological effect by submission to SeattleSeq Annotation 141, and were also checked for continuing presence and frequency data in the latest version of dbSNP, 150. In the 1000 Genomes Browser, the ‘British in England and Scotland (GBR)’ subset was taken as closest to a control for the frequencies in the Irish population, and in the gnomAD database we used the frequency in non-Finnish Europeans.

### CpG islands

CpG islands referred to in this article are those as annotated and defined on the UCSC (University of California, Santa Cruz) Genome Browser (http://genome.ucsc.edu/cgi-bin/hgc?hgsid=726695283_EIGgf4ibtvwGEl62LXUf89J7yTNx&c=chr3&l=75955759&r=75956308&o=75955759&t=75956308&g=cpgIslandExt&i=CpG%3A+41).

## DNA availability

The data are given in the supplementary tables. Any additional information can be supplied upon request.

## Supplementary information

Supplementary information.

Supplementary Figure S1.

Supplementary Tables.
