# Supplementary material for: Investigation of DNA variants specific to ROBO2 Isoform ‘a’ in Irish vesicoureteric reflux patients reveals marked CpG island variation
Source: Sci Rep. 2020 Feb 10;10:2265. doi: 10.1038/s41598-020-58818-6 (PMC7010700; doi:10.1038/s41598-020-58818-6)
Supplement: Supplementary file 1 — Supplementary information. [file 41598_2020_58818_MOESM1_ESM.doc]

**Investigation of DNA variants specific to ROBO2 Isoform ‘a’ in Irish vesicoureteric reflux patients reveals marked CpG island variation**

**John M. Darlow, Mark G. Dobson, Andrew J. Green, Prem Puri and David E. Barton**

**Supplementary information**

**ROBO2a sequence showing positions of PCR primers used and variants found**

Exon 1 and upstream sequence

chr3:75,954,395-75,956,444 hg19

chr3:75,905,244-75,907,293 hg38

ROBO2aPromDelF

ggagaaataaagcttgcctagaataatttcttgagtggagaatgaatgat

ctggatagatttattgacaataaatatatctgtacttattttat**t**aatta

atcttcatgaagagaaatataaaagtcatattctagtagttgataccatg

ggaaaaaattcttatccttttcctaaggaaaaatacatcatgacacaaaa

ROBO2v1_01F

gtaaagttaattaaattacttactttattaattgtctataatttctatgg

gttgaagtaaatttgatactcaatgctatttaat**c**taattctaaaagctt

c.-(1386-1384) DelATC

gaatgagtaat**g**attttttcatgtaaggttagttgaaataa**a**ttatcatt

gattttcaaatttaatattaattagctattgcatattcttaatgggaaac

aagtgtttacaa**g**gccttctttggttgcaaaaagaccatgcccattgtag

c.-1318, ROBO2aPromDelR

acttcttagaatacatc**a**catctggcctcttttttagagtggggaattca

actcatatgtggctggacattgattctgaacattttgaacattatttgtc

tgtctaaactcatactggttgctgttcttttataagtaatcaccatgttt

agcatgaacttgagctattgttacttagggtgcttgactatttacaagtc

ROBO2v1_02F

tcactaggattctaggaattcattcgtcttgtggtgtgtatttgccgata

gtcctattatctttgttggttcaattacacagtac**c**atccaagatgctgc

c.-942

aacacattatacacagggtatgggttgtgagagctaca**g**atttcagttat

tattcaggctcagaaacttagaatctctgcgagctggaacatgttactta

ROBO2v1_01R

gtcttgttttaagaccg**t**cttgttttaaaagtcagtctcaaattcttaaa

atatagaatagggatgctagtagtgctcacagaattgtagtaaagattag

ct**g**gggaaaaaagagactattaaacagagcctggtattcaataagtatca

gttgcctt**c**gattagcaatctgtgttgtcatgattagaagattgctggtt

tatgttacagacctgaaagtatggtcaaaatatgtcaaaataacaaccag

ROBO2v1_03F

acagaatcatataccattccttgaacatttcatccctagaatctagccaa

attagcatctctatacagacaatactgttaacacagatatgtgctcaaac

ROBO2v1_02R, ROBO2v1_04F

ccctcatttacttttcagcacttgtaaattactggagatatttaggaagg

agtctggggttatggcttagttaagctgagtggctagaaagtgtgagatg

ctggatgcctcctccagttcctgtctccagtggccgcacaagg**c**atatcc

c.-365, ROBO2aE1AF

acagctctgctcctg**c**gaactcgtttctggcgtggggcttgtgcgcaccc

c.-320,

c.-231

cagaccgggaggagcctcgagcctgctcattagtagcacgggcagct**c**gc

GCGCTGGAGGAGGGAGGCGGAAGGACAGCGCTGCGCCACCACCCGGAGG**A**

GGGAGCGCGGTAGCT**G**CAGGCAGGGGAGGGAGAGGAAAGAAAAG**G**AAGGA

c.-111, ROBO2aE1AR & ROBO2aE1BF

(same position, opposite strand)

overlapping ROBO2v1_03R,

c.-78, c.-67

CGGCT**C**CCAGACAGAGAGTGGGAGAAACCGGGGAGCAGCGGGAGCAGCAG

GTCCGGGGGGAGCTGTTCCGCTGC**G**CTGCCCTCGTTATTCACACGGACGC

TGCGGAGCTTCCC**A**GGGCTGCTTCCCTGTCCCCCTGGGTGGAGGCTGCCG

TCTAAACCTGACTCCAGgtgagttc**g**gcaaagaggtgaaccgaggggtct

cgaaccagagacgtggcattggcgaggctgctcagctcc**c**ccaagcgctg

CpGendF

ROBO2aE1BR overlapping

ROBO2v1_04R c.-14+127

ctgcctttatatttcccaaattcacggaaggtcttggcggtgg**c**tgcaca

ggctgggagggggcggagggtcggtgggtggttgcgatcctcacggccag

cgtgggggcattcggatggaattagcttaagtgaatggggttttaggaaa

CpGendR

tgtcc**c**caaaggcacgcgttatatatgtgtttctagacaagcttttaaga

cggaaatttggtttcccggatgccctttttggccttctgtccttcctttt

Commercial primers (only parts matching sequence shown), green forward, brown reverse

In-house primers grey

Variants already known in dbSNP at the start of our investigations are shown in purple.

Variants not in dbSNP at the time or rare, and therefore investigated, are shown in cyan.

(c.-111 is the variant still unknown except in our data.)

Other reported SNPs, not found in our samples, are underlined and in bold type. Red type denotes known protein-binding sites.

CpG island shaded in pale yellow

Capital letters indicate mRNA as in NCBI Reference Sequence: NM_001128929.3; GenBank mRNA sequence DQ533873.1, used by the UCSC Genome Browser, has the start 19 bp later, as at the beginning of the next line, *i.e.* GCGCTGGAGG *etc*., and Ensembl 1 nt further (CGC..).

Exon 2

Chr3 seq of Beckman Coulter ROBO2v1_05 amplicon with 1 Kb either side covering ROBO2a Exon 2 (shown in capitals) showing in-house primer positions. The coding starts at the ATG shaded pale blue. All the reported variants are boxed. Only the one shaded in cyan and not within a primer was found to be a real variant on the Chromosome 3 copy of the sequence.

chr3:75,985,370-75,987,904 hg19

chr3:75,936,219-75,938,753 hg38

gtctgaagttaagta**a**tgcaaagttggtaattaaatcttcccctttc**a**tg

atgtaggagaaagggagaaagaaaacttctgg**g**t**g**actggattccctt**g**c

aagtttattagaa**t**tgtaattatg**c**agcattttgaataaagcattcattc

tggaaat**t**atta**ag**tttacttttcttaactccgtattttgtcaactgtgg

aaaaattgggatttagga**g**taacctctgatttaaaattcca**t**ctg**c**taa**c**

catttaaattgtagtcttgtaatttcctaactttggtcttgaaggtgttt

atcattcttatgc**at**tattatactgtatta**t**atatttatgcaacataaaa

tgtatagttttaactattgtac**a**tg**ttt**acatatgaaaggta**t**a**g**ttttg

aactt**atc**attattcaa**t**ttg**c**tagttttgc**t**tcactattcatttttgac

a**ctta**tccatattagtacatgcaac**t**caagtttattcttt**a**gagctactg

t**g**tatcattcaaatgtatgaataaataaccttgctttggtggacatttaa

gtattttcat**a**t**a**tca**a**t**a**tgaaaagcagtttcaaaatgaacattcaaat

atacatt**g**tcatatatatgtataat**a**tttgtatatatgaaa**gt**tcctgtg

ggttatatatctgaaa**t**caaaattacaagtgttttgtaa**tg**tctataatt

aattttact**a**tcaattttaagtaaa**t**aagattagtt**t**ttgcacatgatga

atctctttattcagttaattactg**a**cataattagaat**a**ccacttaaaact

tga**g**aatattttcaa**a**ggtatttatgctaaacttacataa**a**tgtgttgaa

aagtccttttgtatatatgtagagattacatggtatgttaaattcatcaa

ROBO2aE2Chr3F

tttgcctaaatatcaagttatacttaagta**g**tgtccatacacaatttttt

tgcttgactga**actccaa**tatgtatattgggagtactttttaacattcat

ROBO2v1_05F

ggttaaatggcctgttttggaaaatgatattaaaaatgt**g**attcaga**t**ga

atgctt**agaataa**agattaaagatgaatgagctttcatattaatcatcaa

tatgacaatcctaaagggaagcatgattttcaaatgtacctccttgtaag

caggataattcagaaatgca**c**a**a**tgcatattagatatgggatttgagtcg

tagtatatttctctaagaatgtaatttattgtactttcacatccacccca

c**tc**aatatgcagAGTTTAAGATGCAATGGCCAGAAGACATGAA**C**GTGTCA

CTAGAAGGATGTGGAC**A**TGGGCTCC**G**GGACTGTTGATGATGACTGTG**G**T**G**

ROBO2aE2Chr3R

TTTTGGGGTCATCAGGGGAATGGACAAGGCCAAGgtaagtgcaaggatgt

tctaattctttgagagttggatgc**g**aatttcactttatgatga**t**attatg

Position of ROBO2aE2Chr20R

but c = t on Chr20

tgagtctttggtt**c**gacgtttaagcaattt**g**ttgtaat**t**ttc**cc**ccaggt

tttt**g**ttaca**c**ggtattgtaatgcctctgcaccagttaaattgaaataga

**a**tta**t**gtgtgagtaaatgcatcaaaattga**a**gccatctttc**t**gaaattgg

ctttt**a**tctgttggaattgatttt**atatat**at**atatatatatat**atatat

ROBO2v1_05R

atatat**ga**ggtattatgaggcta**t**gtgtgtgtgtgtgtgttttggg**t**ggg

cgggtatgcgt**a**tgagtttgtgt**t**tgtggtatttta**t**aagaacacgtagc

atataaag**ttt**ttgtgattttgaagaaaagtttttggatgtttgttt**a**tt

ttctttagggttttt**g**tgttttacattttaaaatttcaagaa**c**ctattct

taaaggat**g**tgatgttcttt**a**gaaattaaatgaaaaa**t**gtgatgtttctc

attcttccgccaag**t**gtatttaggg**g**aaattaatgatagagtggagctg**t**

aag**g**ttgtggcatcctgaaatgtctgt**t**ggagagtagaaaagggccatag

a**t**tttgtaataaggctttcaata**c**tga**t**ttgatcaatagctgtatggagt

ggtttgtgccaaatctagtatct**c**tga**g**tgtattttgtgataagtattgt

ttttggaggtgtgttggaggttttggaggtgtgttttggagga**t**tattat

ttttggaggtgtgttgcctgctcagatacaaattgcatttccgtatttta

tgtgataaaacatcctgacacc**c**ctgaaattttcttttcct**t**ttggg**g**ca

agtatatttggcattttttc**c**gcatttttaaggcttgaaatgaaatctgt

**a**agca**g**gaagttcacagagtttatttcaagggataggt**g**tatt**a**ct**cc**ag

ataaaattgaaag**tg**tgcagtgttattatataattgtgatgaagttga**g**c

tttg**c**tagtttgaagtatctaataaagtt**t**aacactttttaaaagatcct

tcaaaaag**a**gcagttttcttgccatatgtaatattgttca**a**tgaattttt

gaaatgaaatagatatttttcaaaaccacaa**a**tgc

**Details of PCRs**

The primers for commercial sequencing (for variant discovery) had extensions, not matching the target sequences, that were used for sequencing; the parts matching the targets are shown on the sequences above. The reaction conditions are unknown to us.

In-house PCRs (for variant investigation) were carried out in 10 μl volumes with MyTaq^TM^ DNA Polymerase and MyTaq Buffer (including dNTPs and MgCl_2_) (BioLine, an international company with several national sub-companies), and LCGreen® Plus+ Melting Dye (BioFire Defense, formerly Idaho Technology, Inc, Salt Lake City, Utah, USA) was added for high-resolution melting-curve analysis, using a LightCycler®480. Primers were used at final concentration 0·2 pmol/µl. The sequences and annealing temperatures used are shown in the table below. After an initial melt at 95°C for 1 min, the cycles were 15 seconds each at 95°C, the annealing temperature and 72°C, except for those with the ROBO2v1_04 primers, in which the extension time was 30 seconds. As the reactions could be watched while in progress, the number of cycles could be determined as necessary, and varied between 30 and 65 cycles for the amplifications of upstream and Exon 1 sequences and up to 99 cycles for the amplifications of Exon 2 and its genomic copies.

The reverse primer for the ATC insertion/deletion variant (the ROBO2aPromDel pair) was in a position in which there is no match in the region of homology with chromosome 22 (see Supplementary Figure S1), but the primer pairs covering Exon 1 (ROBO2aE1A and ROBO2aE1B) were in positions of identity or near-identity between the chromosome 3 and Chromosome 22 sequences. Therefore, to avoid amplification from both chromosome-pairs, a first round of PCRs was carried out with the ROBO2v1_04 primers, which were Chromosome-3-specific. These were copies of the commercial primers of the same name, but without the extension sequences used by Beckman Coulter. DNA products from these reactions were then diluted by two serial 1/100 dilutions 0·5 µl added to 49·5 µl TE buffer each time and then 0·5 µl of the second dilution was used in the next round of 10 µl PCRs, so the overall dilution was 1/200,000. The procedure was that wells of two 96-well plates were filled with the TE volumes using a multi-dose pipette and then the serial dilutions from the initial PCRs to the 2^nd^-round PCRs were carried out with multi-channel pipettes.

In-house primers

| Primer-pair name | Sequence | Annealing Temp °C | Variant(s) |
| --- | --- | --- | --- |
| ROBO2aPromDel | F 5′-tgagtggagaatgaatgatctgg-3′ | 60 | c.-1386_-1384del |
|  | R 5′-agtctacaatgggcatggtct-3′ |  |  |
| ROBO2v1_04 | F 5′-AAGTGTGAGATGCTGGATGC-3′ | 64 | 1st round nested |
|  | R 5′-AAGACCTTCCGTGAATTTGG-3′ |  | PCR of E1A & E1B |
| ROBO2aE1A | F 5′-gaactcgtttctggcgtgg-3′ | 60 | c.-320 & c.-111 |
|  | R 5′-CGTGTGAATAACGAGGGCAG-3′ |  | (c.-231 included) |
| ROBO2aE1B | F 5′-CTGCCCTCGTTATTCACACG-3′ | 60 | c.-78 |
|  | R 5′-gaccttccgtgaatttggga-3′ |  | (c.-67 included) |
| ROBO2aCpGend | F 5′-CCAGAGACGTGGCATTGG-3′ | 57 | (c.-14+127 = |
|  | R 5′-CTTGTCTAGAAACACATATATAACGCG-3′ |  | rs182521620) |
| ROBO2aE2Chr3 | F 5′-gggagtactttttaacattcatg-3′ | 60 | Exon 2 |
|  | R 5′-tcgcatccaactctcaaagaa-3′ |  |  |
| ROBO2aE2Chr20 | F 5′-gggagtactttttaacattcatagg-3′ | 60 | Exon 2 copies on |
|  | R 5′-caaattgcttaaacgtcaaacca-3′ |  | Chr20 & Chr22 |

For alignments of the Chromosome 3 sequences with their Chromosome 22 and Chromosome 20 copies, showing positions of the primers, variants and differences between sequence copies, see Supplementary Figure S1. **N.B. Open this file by navigating to it from within WordPad (PCs) or TextEdit or SimpleText (Mac) and setting to ‘No wrap’ in the Word wrap menu in the View menu. Microsoft Word will not display the alignments properly.** WordPad can be found on a PC in the All Programs\Accessories menu. TextEdit is a free downloadable program.

**Details of investigation of Exon 2 and its genomic copies**

Having realised that the commercial primers had co-amplified and sequenced at least one genomic copy of Exon 2, and, at the time, only being aware of the one copy shown in the hg19 build of the Human Genome, on Chromosome 20, we obtained both the *ROBO2a* Exon 2 (Chromosome 3) and matching Chromosome 20 reference sequences with generous margins on either side and aligned them. The homology extends much further from the exon than appeared to be the case for Exon 1, and there was nowhere on either side of the exon that the copies were different enough that we could make completely different chromosome-specific primers for normal short-range PCR. Rather than attempt long-range PCR, we decided to try exploiting a single nucleotide deletion in the Chromosome 3 sequence with respect to the Chromosome 20 sequence. This lies between positions -262 and -263 from the real *ROBO2a* Exon 2 on Chromosome 3. The respective Chromosome 20 sequence contains an inserted adenosine in this position. We found that -263 was the 5′-most nucleotide of the Beckman Coulter forward primer, and the difference at this end of the primer did not prevent it binding equally well to both chromosomes 3 and 20. We made new primers that placed this position just behind the 3′ end of the forward primers thus, Chromosome 3-specific primer gggagtactttttaacattcatg, Chromosome 20-specific primer gggagtactttttaacattcat**A**gg, where the capital ‘A’ indicates the inserted nucleotide in the Chromosome 20 reference sequence.

Because of this difference between the forward primers, the primer-design program that we used (Primer3) chose different reverse primers, giving a 448-bp amplicon for Chromosome 3 and a 503-bp one for Chromosome 20. These amplicons respectively excluded the last 6 and last 4 of the 18 variants reported in the 535-bp commercial amplicon, so there were only 12 within the region covered by both of our hoped chromosome-specific primer pairs, but there were another 7 positions in which we noticed two bases in the chromatograms, making 19 positions in all to be compared. By checking the reported variants for each of our index cases, we chose samples so that for every variant position we had at least three samples that were apparently heterozygous. 39 were chosen out of the 245 originally sequenced.

We amplified all of the 39 selected samples with each of the new primer-pairs, sequenced all 78 products by Sanger sequencing from both ends, and then tabulated the genotypes at each of the 19 positions in each of the 78 sequences (Supplementary Table 1). The results showed that there were 4 positions where the sequence was homozygous for one nucleotide with the Chr3 primers and homozygous for a different nucleotide with the Chr20 primers, one position where the sequence was homozygous for one nucleotide with the Chr20 primers but some samples were homozygous and some heterozygous with the Chr3 primers, 4 positions where the sequence was homozygous for one nucleotide with the Chr3 primers but some samples were homozygous and some heterozygous with the Chr20 primers, and 9 positions where all of the samples were homozygous with the Chr3 primers and all of the samples were heterozygous with the Chr20 primers. These results indicated that (a) the Chr3 primer-pair had successfully amplified only the true chromosome 3 sequence including Exon 2 of *ROBO2a*, and there was only one variant position, which was heterozygous in 13 of the 39 samples sequenced, and (b) the Chr20 primer-pair had not amplified the Chr3 sequence but had undoubtedly amplified at least one other genomic copy in addition to the one already known on Chromosome 20, and probably two, judging from the relative sizes of the alternative peaks in the chromatogram.

Confident that the Chr3 primers were specific, we then used them to screen all the rest of the VUR index cases by PCR and high-resolution melting-curve analysis, including some of the sequenced heterozygotes of the one variable position as positive controls, and then sequenced all PCR products that showed melting-curves that deviated from the rest. We found, when we examined the sequences, that in these reactions we always had a trace of amplification of the other genomic copies, though we had seen no such traces in the sequences of the initial 39 PCRs that had been done immediately after resuspending the lyophilised primers. The follow-up PCRs were done with frozen aliquots of the resuspended primers, and presumably the small amount of genomic-copy contamination was caused by a loss of the 3′ terminal nucleotide from a few molecules of the oligonucleotide forward primer, which would allow it to match to the Chr20 sequence. However, it was easy to exclude from being real any apparent nucleotide variants in the sequences that were due to these traces of other genomic copies, because any such artefacts always occurred in every sequence. The result was that we established that there were no novel variants in the Exon 2 amplicon in any of the 251 index case samples. There was just the one variant that we had found in the initial sequencing. It is in Intron 1 at -64 relative to the start of the exon, and there were 17 samples heterozygous for it, most of which had been picked out as heterozygous in the commercial sequencing and been included in the initial set of 39 that we sequenced. This variant was initially assigned the identity rs138688911 in dbSNP, and not assigned to either Chr3 or Chr20, as there was some doubt. It is now assigned to Chr3 only, and has the identity rs539728114. The allele frequency in our VUR patients corresponds very closely to the UK population frequency (Table 1), indicating that it is not associated with VUR.

As for the sequences amplified by the Chr20 primer-pair, these of course are not directly relevant to *ROBO2*, but one obviously needs to be aware of them as interference in investigating *ROBO2a*, and we were intrigued by discovering that there were extra copies that were not present in genome build GRCh37/hg19. We tried submitting the Chr20 reference sequence of the Exon 2 amplicon and its surroundings to BLAT in the latest build of the human genome, GRCh38/hg38, and this showed two additional matches in addition to those on chromosomes 20 and 3. These were both on Chromosome 22, one assigned to a particular location and the other still unlocated within the chromosome 22 map. We obtained the reference sequences and aligned all four copies. The three non-Chr3 sequences were identical at the places where we had our forward and reverse Chr20 primers. We marked all the positions of differences between the copies. These differences accounted for most of the apparent variants seen in the products of amplification with our Chr20 primers (though two appear to be genuine SNPs) and revealed another two that we had not noticed. They are close to the forward primer and one of them can only be seen in the reverse sequences. The latter, at the position numbered 8 in Supplementary Fig. S1, is within the binding-site of the commercial sequencing company’s forward primer, ROBO2v1_05F, but it is 12 nucleotides back from the matching-place of the 3′ nucleotide of the primer and does not seem to have had much effect upon binding, because the Chromosome 20 sequence clearly was amplified. Within the span of our Chr20 amplicon, there are thirteen base-substitutions in the reference sequences relative to the Chr3 reference sequence. Nine of these are on the Chr20 copy only, two are in the unassigned Chr22 copy only, one is in the assigned Chr22 copy only, and one in both Chr22 copies. This indicates that the initial Chr22 copy has occurred from Chr3 evolutionarily much more recently than the Chr20 copy, and suggests that the two Chr22 copies may have occurred by duplication of one from the other. In addition, a 7-bp insertion-deletion polymorphism is reported in dbSNP in both the Chr3 (rs142488575) and Chr20 (rs200119310) copies, where the insertion allele is the reference sequence for both chromosomes. However, the reference sequences of the Chr22 copies have the ‘insert’ (invariant) in the unassigned copy, but the deletion invariant in the assigned copy. None of our sequences showed any deletion on Chromosome 3 (nor on Chr20). Most sequences amplified with the Chr20 primers appeared to show a heterozygous deletion but this was undoubtedly an artefact due to the presence of the shorter Chr22 copy, which sometimes was poorly amplified. The alignments of all the copies with both Exons 1 and 2 of *ROBO2a* are given in Supplementary Figure S1, which shows the positions of all the variants (see also Supplementary Table 1 and its legend). We also note that the assigned copy of Exon 2 on Chr22 does not represent part of a copy of the whole of the beginning of *ROBO2a*, because it is over 6 mb from the Chr22 copy of Exon 1 and is on the opposite strand.

1 Koressaar, T. & Remm, M. Enhancements and modifications of primer design program Primer3. *Bioinformatics* 2007;23:1289-91.

2 Untergasser, A., Cutcutache, I., Koressaar, T., Ye, J., Faircloth, B. C., Remm, M. & Rozen, S. G. Primer3--new capabilities and interfaces. *Nucleic Acids Res* 2012;40:e115.

Legends to supplementary figure and tables:

**Supplementary Figure S1**

Alignments of the *ROBO2a* Exon 1and Exon 2 sequences and their contexts with their genomic copies, showing the positions and names of primers used for investigation and the positions of differences between reference sequences and of genuine variants. All annotations and explanations are in the file. **N.B. This file will not display properly with Microsoft Word. On a PC, open it with WordPad (not NotePad) and set to ‘No wrap’ in the Word wrap menu of the View menu. On a Mackintosh machine, open with SimpleText or TextEdit (both freely downloadable programs).**

**Supplementary Table S1**

Genotypes in the Exon 2 amplicon at all apparent variant positions in each of 39 VUR index-case samples amplified with each of two primer pairs, one specific for the true *ROBO2a* sequence on Chromosome 3 (Chr3) and the other amplifying only the three copies on Chromosomes 20 and 22 (lines labelled ‘Chr20’). Variant positions (‘Beckman Coulter No.’) heading the columns are numbered from the 5′ base of the (commercial) ‘ROBO2v1_05’ primer, and are the same as those marked in the sequence alignment, bottom set in Supplementary Fig. S1. The rs numbers heading columns are the ones currently in dbSNP (version 150) if the variant is currently a recognised SNP on either Chr3 or Chr20. No SNPs are currently assigned to the genomic copies on Chromosome 22. The three in red type are classified as ‘Suspected’.

To view only the Chr3 genotypes or only the Chr20 genotypes of the samples, go to the tab at the top of Column B and untick Chr20 or Chr3 respectively. Do not untick ‘Select All’, otherwise the headings will disappear except for the very top line, and the notes at the bottom of the table will also be hidden.

Columns with green shading are invariant on Chr3 and invariant with a different base in the genomic copies, but have the same base in all three copies. Columns with pink shading are invariant on Chr3 and are apparently heterozygous in every sample using the Chr20 primers. This, as can be seen from the alignment in Supplementary Fig. S1, is because one or two copies have the same nucleotide as the Chr3 copy but at least one of the genomic copies has a different base homozygously. Blue shading indicates that some samples are heterozygous and some are homozygous reference sequence at the same position on Chr3, the heterozygous ones being shaded. Yellow shading indicates that some samples appear to be heterozygous and some are homozygous reference sequence at the same position in the sequence obtained from PCRs with the Chr20 primer-pair, the apparently heterozygous ones being shaded. In two cases (positions 171 and 216) this appears to be due to genuine SNPs on the Chromosome 20 copy, as the reference bases are the same in all copies. However in the other three such positions the absence of a base in the reference sequence of one of the Chr22 copies appears to be because that copy was amplified very little, if at all, in the particular sample. This suggests that though all three copies on chromosomes 20 & 22 have identical reference sequences in the positions of the forward and reverse primers, there may in fact be SNPs on both Chr22 copies in one or other of the primer sequences that affect the relative amplification of the copies.

**Supplementary Table S2**

Genotypes and numbers of CpG dinucleotides at each of the six consecutive variable sites in the *ROBO2a* CpG island in each of the 251 VUR index cases, and in the family members of the case with the novel variant (at position c.-111) with haplotype deductions for the family. The numbers in the first column are the sample-numbers. The columns headed ‘R’ and ‘V’ (standing for ‘reference’ and ‘variant’) do not necessarily contain a reference or variant allele on every line, because of course many samples are homozygous at any particular position, but, where there is heterozygosity, the labels indicate which allele is the reference and which is the variant.
