# Supplementary material for: Investigation of DNA variants specific to ROBO2 Isoform ‘a’ in Irish vesicoureteric reflux patients reveals marked CpG island variation
Source: Sci Rep. 2020 Feb 10;10:2265. doi: 10.1038/s41598-020-58818-6 (PMC7010700; doi:10.1038/s41598-020-58818-6)
Supplement: Supplementary file 2 — Supplementary Figure S1. [file 41598_2020_58818_MOESM2_ESM.rtf]

Supplementary Figure S1. 
N.B. if you have opened this file with Microsoft Word, close it and open it with WordPad, or SimpleText or TextEdit (Macintosh machines) and set to 'No wrap' in the View menu.


Exon 1 and upstream sequence  showing matches to Chromosome 22 sequence  Exon in CAPITAL letters

In some places, spaces have been inserted for alignment, and in some places spaces have not been inserted, as seemed appropriate for clarity.
Primer positions, and positions of variants detected on the Chromosome 3 sequence in our index cases, are shaded (or marked above the sequence) with the same colours as in the Supplementary Information.
Primers with names beginning 'ROBO2v1' are those used by Beckton Dickinson for variant discovery.
Nucleotides underlined and in bold are reported as SNPs in dbSNP but were not different from the reference sequence in our samples unless also marked with shading.
Base-substitution differences in the Chromosome 22 sequence within primer matches are shaded in red. (Text in red = binding-site matches.)                                                                                                                                                                                                                                                                                                                                                                                                                                                                                                                                                                                                                                                                                                                                                                                                                                                                                                                                                                                                                                                                                                                                                                                                                                                                                                            ┌--- CpG island -> ----------------------------------------------------------------------------------------------------------- CpG island -------------------------------------------------------------------------------------------------------- CpG island ------------------------------------------------------------------------------------------------------------------------------------- CpG island --------------------------------------------------------------------------------------------------------------------------------------- <- CpG island -┐
                                                                                                                                                                                                                                                                                                                                                                                                                                                                                    ROBO2aPromDelR                                                                                                                                                                                                                                                                                                                                                                                       ROBO2v1_01R                                                                                                                                                                                                                                                                                                                                                                                                                                <- ROBO2v1_02R                                                                                                    |                                                                                                                                                                                                                                                                      ROBO2aE1BF              c.-78      c.-67                                                                                                                                                   ROBO2aE1BR complement                                                                               |
                                                                                                                                                                                                                                                                                                  ROBO2v1_01F                                                                                                                                             c.-1318                     complement                                                                                                                                                                                                                                        ROBO2v1_02F                                                                                                c.-942                                complement                                                                                                                                                                                                                                                                                            ROBO2v1_03F                                                                                                                             complement                           ROBO2v1_04F ->                                                         c.-365  ROBO2aE1AF                           c.-320                                                                                    c.-231                                                                                                                   c.-111    ROBO2aE1AR   ROBO2v1_03R complements                                                                                              ROBO2aCpGendF                                              ROBO2v1_04R complement  c.-14+127                                                                 |                                                         ROBO2aCpGendR complement
These are hg19 co-ordinates                                     ROBO2aPromDelF                                                                                                                                                                                                           1      10|       20|       30|       40|       50|       60|       70|       80|       90|      100|      110|      120|      130|      140|      150|      160|      170|      180|      190|      200|      210|      220|      230|      240|      250|      260|      270|      280|      290|      300|      310|      320|      330|      340|      350|      360|      370|      380|      390|      400|      410|      420|      430|      440|      450|      460|      470|      480|      490|      500|      510|      520|      530|      540|      550|      560|      570|      580|      590|      600|      610|      620|      630|      640|      650|      660|      670|      680|      690|      700|      710|      720|      730|      740|      750|      760|      770|      780|      790|      800|      810|      820|      830|      840|      850|      860|      870|      880|      890|      900|      910|      920|      930|      940|      950|      960|      970|      980|      990|     1000|     1010|     1020|     1030|     1040|     1050|     1060|     1070|     1080|     1090|     1100|     1110|     1120|     1130|     1140|     1150|     1160|     1170|     1180|     1190|     1200|      1210|     1220|     1230|     1240|     1250|     1260|     1270|     1280|     1290|     1300|     1310|     1320|     1330|     1340|     1350|      1360|     1370|     1380|     1390|     1400|     1410|     1420|     1430|     1440|     1450|     1460|     1470|     1480|     1490|     1500|     1510|     1520|     1530|     1540|     1550|     1560|     1570|     1580|     1590|     1600|     1610|     1620|     1630|     1640|     1650|     1660|     1670|     1680|     1690|     1700|     1710|     1720|     1730|     1740|     1750|
>chr3:75,954,395-75,956,594  ggagaaataaagcttgcctagaataatttcttgagtggagaatgaatgatctggatagatttattgacaataaatatatctgtacttattttattaa    ttaatcttcatgaagagaaatataaaagtcatattctagtagttgataccatgggaaaaaattcttatccttttcctaaggaaaaatacatcatgacacaaaagtaaagttaattaaattacttactttattaattgtctataatttctatgggttgaagtaaatttgatactcaatgctatttaatctaattctaaaagcttgaatgagtaatgattttttcatgtaaggttagttgaaataaattatcattgattttcaaatttaatattaattagctattgcatattcttaatgggaaacaagtgtttacaaggccttctttggttgcaaaaagaccatgcccattgtagacttcttagaatacatcacatctggcctcttttttagagtggggaattcaactcatatgtggctggacattgattctgaacattttgaacattatttgtctgtctaaactcatactggttgctgttcttttataagtaatcaccatgtttagcatgaacttgagctattgttacttagggtgcttgactatttacaagtctcactaggattctaggaattcattcgtcttgtggtgtgtatttgccgatagtcctattatctttgttggttcaattacacagtaccatccaagatgctgcaacacattatacacagggtatgggttgtgagagctacagatttcagttattattcaggctcagaaacttagaatctctgcgagctggaacatgttacttagtcttgttttaagaccgtcttgttttaaaagtcagtctcaaattcttaaaatatagaatagggatgctagtagtgctcacagaattgtagtaaagattagctggggaaaaaagagactattaaacagagcctggtattcaataagtatcagttgccttcgattagcaatctgtgttgtcatgattagaagattgctggtttatgttacagacctgaaagtatggtcaaaatatgtcaaaataacaaccagacagaatcatataccattccttgaacatttcatccctagaatctagccaaattagcatctctatacagacaatactgttaacacagatatgtgctcaaacccctcatttacttttcagcacttgtaaattactggagatatttaggaaggagtctggggttatggcttagttaagctgagtggctagaaagtgtgagatgctggatgcctcctccagttcctgtctccagtggccgcacaaggcatatccacagctctgctcctgcgaactcgtttctggcgtggggcttgtgcgcaccccagaccgggaggagcctcgagcctgctcattagtagcacgggcagctcgcgCG CTGGAGGAGGGAGGCGGAAGGACAGCGCTGCGCCACCACCCGGAGGAGGGAGCGCGGTAGCTGCAGGCAGGGGAGGGAGAGGAAAGAAAAGGAAGGACGGCTCCCAGACAGAGAGTGGGAGAAACCGGGGAGCAGCGGGAGCAGCAGGTCC GGGGGGAGCTGTTCCGCTGCGCTGCCCTCGTTATTCACACGGACGCTGCGGAGCTTCCCAGGGCTGCTTCCCTGTCCCCCTGGGTGGAGGCTGCCGTCTAAACCTGACTCCAGgtgagttcggcaaagaggtgaaccgaggggtctcgaaccagagacgtggcattggcgaggctgctcagctcccccaagcgctgctgcctttatatttcccaaattcacggaaggtcttggcggtggctgcacaggctgggagggggcggagggtcggtgggtggttgcgatcctcacggccagcgtgggggcattcggatggaattagcttaagtgaatggggttttaggaaatgtccccaaaggcacgcgttatatatgtgtttctagacaagcttttaagacggaaatttggtttcccggatgccctttttggccttctgtccttccttttgcttgcctcatgcccaattttccaagccaaggaaaggtttgggaaggcgtggaagcagaaggtctgggcacagaatgtgacggggcaaccttggtctctaagtttgggggtgggggttgggactctt  cctttccacctaatttaccaggg
>chr22:17,198,328-17,200,079 ggagaaataaagcttgcctagaataatttcttgagtggagaatgaatgatctggatagatttgttgacaataaatatgtctgtacttaatttattaattctttaatcttcatgaagaaaaatataaaagtcatattctagtattttataccatggaaaaaaattcttatctttttcctaaggaaaa                                                                            No match                      This section is deleted with respect to the Chr3 copy  i.e. chr3:75,954,577-75,955,030 hg19 (454 bp) has no match on Chromosome 22.                   No match                                                                                                                                                                                           actatttacaagtctcactaggagtctaggtattcattagtctcgtggtgtgtatttgctgatagtcctgttatctttgctggttcaattacacagtaccatccaagacgctgcaacacgttatacacagggtatgggttgtgagagctacagatttcagttattattcaggctcagaaacttagaatctctgcgagctggaacatgttacttagtcttgttttaagaccgtcttgttttaaaagttagtctcaaattcttaaaatatagaatagggatgctagtagtgctcacagaattgtagtaaagattagctgggaaaaaagagactattaaacagagcctggtattcaataagtatcagttgccttccattagcaatctgtgttgtcatgattagaagattgctggtttatgttacagatctgtaagtacagtcaaaatagcaaccagacaaaatcatgtaccattccttcaacatttcatccctagaatctagccaaattagcatctctatacaaacaataatgttaacacagatatgtgctcaaacccctcatttacttttcagcacttgtaagttacgggagagatttaggaagaagtctggggttatggcttagttaagctgagtggctagaaagtgtgagatgctagaggggtacagatggttgcctcctccagttcatttctccagtggccgcacaaggcatatccatagctctgctcctgggaactcgtttctggcgtggggcttgtgcgcaccccagaccgggaggagcctggagcctgctcattagtagcacgggcagctcctgcgcctggaggagggaggcggaaggacagccctgcgccaccaccaggagaagggagcgcggtagctgcaggcaggggagggagaggaaagaaaaggaaagacggctcccagacagagagtggaagaaatcggggagcagccggagcagcaggtccgggggggagctgttccgctgcgctgcgctcattattcacacggacgctgcggagcttcccagggttgcttcccagtccccctgggtggaggctgtcgtctaaacctgactccaggtgagttcggcaaagaggtgaacggaggggtctcgaaccagagacgtggaattggcgaggctgctcagctcccccaagcactgctgcctttatatttcccaaattcacggaaggttttggcggtagctgcacagtctgcgagtgggcggatgggcggtgggtggttgcgatcctcacagccagcatggaggcattcggatggaattagcttaagtgaatggggttttaggaaatgtccccaaaggcaagcattatgtatgtgtttctagacaagcttttaagacagaaatttggtttcccggatgcccattttggccttctgtccttccttttgcttgcctcatgcccaattttccaagccaaggaaaggattgggaaggcgtggaagtagaaggtctgggcacagaatgtgacaaggcaaccttggtctctaagtttgggggtgggagttgggactcttttcctttcaacttaatttac
(reverse complement)                                                                                                                                                                                                   %This nucleotide is immediately adjacent to →-----------------------------------------------------------------------------------------------------------------------------------------------------------------------------------------------------------------------------------------------------------------------------------------------------------------------------------------------------------------------------------------------------this nucleotide&                                                                                                                                                                                                                                                                                                                                                                                                                                                                                                                                                                                                                                                                                                                                                                perfect match


Exon 2 and flanking sequence  showing matches to Chromosome 20

Co-ordinates are for hg19 (GRCh37) and include 1,000 bp either side of the Beckman Coulter amplicon, as in the Supplementary Information file.
This shows the information available at the time of planning the sequencing.
In this alignment, spaces have been inserted in both sequences wherever necessary to maintain exact alignment.
Mauve shading above the sequences indicates places where the software used by Beckman Coulter reported variants, supposedly in the ROBO2a sequence.
                                                                                                                                                                                                                                                                                                                                                                                                                                                                                                                                                                                                                                                                                                                                                                                                                                                                                                                                                                                                                                                                                             ↓This is the 5' base of ROBO2v1_05F AND the 3' base of ROBO2aE2Chr3F
                                                                                                                                                                                                                                                                                                                                                                                                                                                                                                                                                                                                                                                                                                                                                                                                                                                                                                                                                                                                                                                                                             ↓   ROBO2v1_05F                                                                                                                                                                                                                                                                                                                                                                                                          ROBO2aE2Chr3R                                                                                                 ROBO2v1_05R
Beckman Coulter positions with variants marked (all in dbSNP). The base numbers correspond to those in Supplementary Table 1.                                                                                                                                                                                                                                                                                                                                                                                                                                                                                                                                                                                                                                                                                                                                                                                                                                                                                                                                              ROBO2aE2Chr3F     1      10|       20|       30|       40|       50|       60|       70|       80|       90|      100|      110|      120|      130|      140|      150|      160|      170|      180|      190|      200|      210|      220|      230|      240|      250|      260|      270|      280|      290|      300|      310|      320|      330|      340|      350|      360|      370|      380|      390|      400|      410|      420|      430|      440|      450|      460|      470|      480|      490|      500|      510|      520|      530|     
>chr3:75,985,370-75,987,904                         gtctgaagttaagtaatgcaaagttggtaattaaatcttcccctttcatgatgtaggagaaagggagaaagaaaacttctgggtgactggattcccttgcaagtttattagaattgtaattatgcagcattttgaataaagcattcattctggaaattattaagtttacttttcttaactccgtattttgtcaactgtggaaaaattgggatttaggagtaacctctgatttaaaattccatctgctaaccatttaaattgtagtcttgtaatttcctaactttggtcttgaaggtgtttatcattcttatgcattattatactgtattatatatttatgcaacataaaatgtatagttttaactattgtacatgtttacatatgaaaggtatagttttgaacttatcattattcaatttgctagttttgcttcactattcatttttgacacttatccatattagtacatgcaactcaagtttattctttagagctactgtgtatcattcaaatgtatgaataaataaccttgctttggtggacatttaagtattttcatatatcaatatgaaaagcagtttcaaaatgaacattcaaatatacattgtcatatatatgtataatatttgtatatatgaaagttcctgtgggttatatatctgaaatcaaaattacaagtgttttgtaatgtctataattaattttactatcaattttaagtaaataagattagtttttgcacatgatgaatctctttattcagttaattactgacataattagaataccacttaaaacttgagaatattttcaaaggtatttatgctaaacttacataaatgtgttgaaaagtccttttgtatatatgtagagattacatggtatgttaaattcatcaatttgcctaaatatcaagttatacttaagtagtgtccatacacaatttttttgcttgactgaactccaatatgtatattgggagtactttttaacattcat ggttaaatggcctgttttggaaaatgatattaaaaatgtgattcagatgaatgcttagaataaagattaaagatgaatgagctttcatattaatcatcaatatgacaatcctaaagggaagcatgattttcaaatgtacctccttgtaagcaggataattcagaaatgcacaatgcatattagatatgggatttgagtcgtagtatatttctctaagaatgtaatttattgtactttcacatccaccccactcaatatgcagAGTTTAAGATGCAATGGCCAGAAGACATGAACGTGTCACTAGAAGGATGTGGACATGGGCTCCGGGACTGTTGATGATGACTGTGGTGTTTTGGGGTCATCAGGGGAATGGACAAGGCCAAGgtaagtgcaaggatgttctaattctttgagagttggatgcgaatttcactttatgatgatattatgtgagtctttggttcgacgtttaagcaatttgttgtaattttcccccaggtttttgttacacggtattgtaatgcctctgcaccagttaaattgaaatagaattatgtgtgagtaaatgcatcaaaattgaagccatctttctgaaattggcttttatctgttggaattgattttatatatatatatatatatatatatatatatatgaggtattatgaggctatgtgtgtgtgtgtgtg        ttttgggtgggcgggtatgcgtatgagtttgtgtttgtggtattttataagaacacgtagcatataaagtttttgtgattttgaagaaaagtttttggatgtttgtttattttctttagggtttttgtgttttacattttaaaatttcaagaacctattcttaaaggatgtgatgttctttagaaattaaatgaaaaatgtgatgtttctcattcttccgccaagtgtatttaggggaaattaatgatagagtggagctgtaaggttgtggcatcctgaaatgtctgttggagagtagaaaagggccatagattttgtaataaggctttcaatactgatttgatcaatagctgtatggagtggtttgtgccaaatctagtatctctgagtgtattttgtgataagtattg     tttttggaggtgtgttggaggttttggaggtgtgttttggaggattattatttttggaggtgtgttgcctgctcagatacaaattgcatttccgtattttatgtgataaaacatcctgacacccctgaaattttcttttccttttggggcaagtatatttggcatt  ttttccgcatttttaaggcttgaaatgaaatctgtaagcaggaagttcacagagtttatttcaagggataggtgtattactccagataaaattgaaagtgtgcagtgttattatataattgtgatgaagttgagctttgctagtttgaagtatctaataaagtttaacactttttaaaagatccttcaaaaagagcagttttcttgccatatgtaatattgttcaatgaatttttgaaatgaaatagatatttttcaaaaccacaaatgc
>chr20:26,212,571-26,210,037                   tatgagtctgaagttaagtaatgcaaagttgttaattaaatcttccccttttgtgatgtaggagaaagggagaaagaaaacttctgggtgactggattcccttgcaagtttattagaattgtaattatgtagcattttgaataaagcattcattctggaaatcattaagtttacttttctgagctccgtattttgtcaactgtagaaaaattgggatttaggagtaacctctgatttaaaattccacttgttaatcatttaaattgtagtcttgtaatttcctaactttggtcttgaaggtgtttatcattcttatgcgttattatgctgtattatatatttatgcaacataaaatgtatagttttaactattgtacatgtttacatatgaaaggtatagttttgaacttatcattattcagtttgctagttttgcttcactattcatttttgacacttatccatattagtacatgcaactcaagtttattcttttgatctagtgtatatcattcaaatgtatgaataaataaccttgctttggtggacatttaagtattttcatgtgtcagtatgaagagcagtttcaaaatgaacattcaaatatacattgtcatatata      atatttgtatatatgaaagttcctgtgggttatatctctgaaatcaaaactacaagtgttttgtaacttctataattaattttactctcaattttaagtaaacaagattagcttttgtacatgatgagtctctttattcagttaattactgacataattagaataccacttaaaacttgagaatattttcaagggtatttatgctaaacttacagaagtgtgttgaaaagtccttttgtatatatgtagagattacatggtatgttaaattcattaatttgcctaaatatcaagttatacttaagtagtgtccatacacaacttttttgcttgactgaactccaatatgtatattgggagtactttttaacattcataggttaaaaggcctgttttggaaaatgatattaaaaatgtaattcagatgaatgcttagaataaagattaaagatgaatgagctttcatattaatcatcaatatgacaatcctaaagggaagcatgattttcaaatgtacctccttgtaagcaggataattcagaaatgcacaatgcatattagatatgggatttgagtcgtaatatatttctctaagaatgtaatttattgtactttcacatccaccccactgaatatgcagagtttaagacgcgatggccagaagacatgaaagtgtcactagaaggatgtggacgtgggctctgggactgttgatgatgactgtggtcttttggggtcatcaggggaatggacaaggccaagacaagtgcaaggatgttctaattctttgagagttagatggaaatttcactttatgatgaaattatgtgagtctttggtttgacgtttaagcaatttgttgtaatcttctgccaggtttttgttacagggtattgtaatgcctctgcaccagttaaattgaaatagaattaggtgtgagtaaatgcatcaaaattgaaggcatctttctgaaattggcttttctctgttggaattgat        tatatatatatatatatatgtatgtgtgaggtat   gaggctatatgtgtgtgtgtgtgtgtgtgtgttttgggtgggtgggtatgcatgtgagtttgtgtgtgtggtattttacaagaacacgtagcatataaagttgttgtgattttgaagaaaagtttttggatgtttgtttcttttcattagggtttttaaattttacattttaaaatttcaagaacctattcttaaaggatgtgatgttctttagaaattaaatgaaaaatgtgacgtttctcattcttctgccaagtgtatttaggggaaattaatgatagagtggaactgcaaggttgtggcatcctgaaatgtctgttggagagtagaaaagggccatagattttgtaataaggctttcaatactgatttgatcaatagctgtatggagtggtttgtgccaaatctagtatctgtgaatgtattttgtgataagtattatattatttttggaggtgtgttg                                                  cttgctcagatacaaattgcaattctgtattttatgtgataaaacatcctgacacccctgaaattttcttttccttttggggcaagtatatttggcattgattttctgcattttcaaggcttgaaatgaaatctgtgagcaggaagttcacagagtttatttcaagggataggtatatttctccagataaaattgaaagtgtgcagtgttattatataattgtgatgaagttgagctttgctagtttgaagtatctaataaagtttaacactttttaaaagatccttcaaaaagagcagttttcttgccatatgtaatattgttcagtgaatttttgaaatgaaatagatatttttcaaaaccacaaatgcatctgtgcctttgtatatgaatagttgtatatacatgtaagcagat
(i.e. reverse complement)                                                                                                                                                                                                                                                                                                                                                                                                                                                                                                                                                                                                                                                                                                                                                                                                                                                                                                                                                                                                                                                  ROBO2aE2Chr20F   ­↑This nucleotide is an insertion with respect to the genuine ROBO2 sequence on Chromosome 3.                                                                                                                                                                                                                                                                                                                                                                                   ROBO2aE2Chr20R


Alignments of ROBO2a Exon 2 and sequences of genomic copies from hg38 (GRCh38) Scroll across to the exon and its copies.

Within the amplified sequence, Nucleotides in the Chromosome 3 sequence that are different from those in all the copies are shaded in green.
                               Nucleotides in each copy sequence that are different from those in the other copies are shaded in mauve.

Beckman Coulter position number:                                                                                                                                                                                                                                                                                                                                                                                                                                                                                                                                                                                                                                                                                                                                                                                                                                                                                                                                                                                                                                                         1     8|                   30|       40|     48|        |57-63|                                                                                                        171| |173                   199|203|         216|                                 253|               272|  |275            294|                   317|    325||326               348| |350                            385||386                          419| 424||425            444|                464|
>chr3:75,936,219-75,938,753                        gtctgaagttaagtaatgcaaagttggtaattaaatcttcccctttcatgatgtaggagaaagggagaaagaaaacttctgggtgactggattcccttgcaagtttattagaattgtaattatgcagcattttgaataaagcattcattctggaaattattaagtttacttttcttaactccgtattttgtcaactgtggaaaaattgggatttaggagtaacctctgatttaaaattccatctgctaaccatttaaattgtagtcttgtaatttcctaactttggtcttgaaggtgtttatcattcttatgcattattatactgtattatatatttatgcaacataaaatgtatagttttaactattgtacatgtttacatatgaaaggtatagttttgaacttatcattattcaatttgctagttttgcttcactattcatttttgacacttatccatattagtacatgcaactcaagtttattctttagagctactgtgtatcattcaaatgtatgaataaataaccttgctttggtggacatttaagtattttcatatatcaatatgaaaagcagtttcaaaatgaacattcaaatatacattgtcatatatatgtataatatttgtatatatgaaagttcctgtgggttatatatctgaaatcaaaattacaagtgttttgtaatgtctataattaattttactatcaattttaagtaaataagattagtttttgcacatgatgaatctctttattcagttaattactgacataattagaataccacttaaaacttgagaatattttcaaaggtatttatgctaaacttacataaatgtgttgaaaagtccttttgtatatatgtagagattacatggtatgttaaattcatcaattt gcctaaatatcaagttatacttaagtagtgtccatacacaatttttttgcttgactgaactccaatatgtatattgggagtactttttaacattcat ggttaaatggcctgttttggaaaatgatattaaaaatgtgattcagatgaatgcttagaataaagattaaagatgaatgagctttcatattaatcatcaatatgacaatcctaaagggaagcatgattttcaaatgtacctccttgtaagcaggataattcagaaatgcacaatgcatattagatatgggatttgagtcgtagtatatttctctaagaatgtaatttattgtactttcacatccaccccactcaatatgcagagtttaagatgcaatggccagaagacatgaacgtgtcactagaaggatgtggacatgggctccgggactgttgatgatgactgtggtgttttggggtcatcaggggaatggacaaggccaaggtaagtgcaaggatgttctaattctttgagagttggatgcgaatttcactttatgatgatattatgtgagtctttggttcgacgtttaagcaatttgttgtaattttcccccaggtttttgttacacggtattgtaatgcctctgcaccagttaaattgaaatagaattatgtgtgagtaaatgcatcaaaattgaagccatctttctgaaattggcttttatctgttggaattgattttatatatatatatatatatatatatatatatatgaggtattatgaggctatgtgtgtgtgtgtgtgttttgggtgggcgggtatgcgtatgagtttgtgtttgtggtattttataagaacacgtagcatataaagtttttgtgattttgaagaaaagtttttggatgtttgtttattttctttagggtttttgtgttttacattttaaaatttcaagaacctattcttaaaggatgtgatgttctttagaaattaaatgaaaaatgtgatgtttctcattcttccgccaagtgtatttaggggaaattaatgatagagtggagctgtaaggttgtggcatcctgaaatgtctgttggagagtagaaaagggccatagattttgtaataaggctttcaatactgatttgatcaatagctgtatggagtggtttgtgccaaatctagtatctctgagtgtattttgtgataagtattgtttttggaggtgtgttggaggttttggaggtgtgttttggaggattattatttttggaggtgtgttgcctgctcagatacaaattgcatttccgtattttatgtgataaaacatcctgacacccctgaaattttcttttccttttggggcaagtatatttggcatt  ttttccgcatttttaaggcttgaaatgaaatctgtaagcaggaagttcacagagtttatttcaagggataggtgtattactccagataaaattgaaagtgtgcagtgttattatataattgtgatgaagttgagctttgctagtttgaagtatctaataaagtttaacactttttaaaagatccttcaaaaagagcagttttcttgccatatgtaatattgttcaatgaatttttgaaatgaaatagatatttttcaaaaccacaaatgc
>chr20:26,229,447-26,231,930 (reverse complement)  gtctgaagttaagtaatgcaaagttgttaattaaatcttccccttttgtgatgtaggagaaagggagaaagaaaacttctgggtgactggattcccttgcaagtttattagaattgtaattatgtagcattttgaataaagcattcattctggaaatcattaagtttacttttctgagctccgtattttgtcaactgtagaaaaattgggatttaggagtaacctctgatttaaaattccacttgttaatcatttaaattgtagtcttgtaatttcctaactttggtcttgaaggtgtttatcattcttatgcgttattatgctgtattatatatttatgcaacataaaatgtatagttttaactattgtacatgtttacatatgaaaggtatagttttgaacttatcattattcagtttgctagttttgcttcactattcatttttgacacttatccatattagtacatgcaactcaagtttattcttttgatctagtgtatatcattcaaatgtatgaataaataaccttgctttggtggacatttaagtattttcatgtgtcagtatgaagagcagtttcaaaatgaacattcaaatatacattgtcata      tataatatttgtatatatgaaagttcctgtgggttatatctctgaaatcaaaactacaagtgttttgtaacttctataattaattttactctcaattttaagtaaacaagattagcttttgtacatgatgagtctctttattcagttaattactgacataattagaataccacttaaaacttgagaatattttcaagggtatttatgctaaacttacagaagtgtgttgaaaagtccttttgtatatatgtagagattacatggtatgttaaattcattaattt gcctaaatatcaagttatacttaagtagtgtccatacacaacttttttgcttgactgaactccaatatgtatattgggagtactttttaacattcataggttaaaaggcctgttttggaaaatgatattaaaaatgtaattcagatgaatgcttagaataaagattaaagatgaatgagctttcatattaatcatcaatatgacaatcctaaagggaagcatgattttcaaatgtacctccttgtaagcaggataattcagaaatgcacaatgcatattagatatgggatttgagtcgtaatatatttctctaagaatgtaatttattgtactttcacatccaccccactgaatatgcagagtttaagacgcgatggccagaagacatgaaagtgtcactagaaggatgtggacgtgggctctgggactgttgatgatgactgtggtcttttggggtcatcaggggaatggacaaggccaagacaagtgcaaggatgttctaattctttgagagttagatggaaatttcactttatgatgaaattatgtgagtctttggtttgacgtttaagcaatttgttgtaatcttctgccaggtttttgttacagggtattgtaatgcctctgcaccagttaaattgaaatagaattaggtgtgagtaaatgcatcaaaattgaaggcatctttctgaaattggcttttctctgttggaattgattatatatatatatatatatgtatgtgtgagg tatgaggctatatgtg  tgtgtgtgtgtgtgtgtgttttgggtgggtgggtatgcatgtgagtttgtgtgtgtggtattttacaagaacacgtagcatataaagttgttgtgattttgaagaaaagtttttggatgtttgtttcttttcattagggtttttaaattttacattttaaaatttcaagaacctattcttaaaggatgtgatgttctttagaaattaaatgaaaaatgtgacgtttctcattcttctgccaagtgtatttaggggaaattaatgatagagtggaactgcaaggttgtggcatcctgaaatgtctgttggagagtagaaaagggccatagattttgtaataaggctttcaatactgatttgatcaatagctgtatggagtggtttgtgccaaatctagtatctgtgaatgtattttgtgataagtatta                                             tattatttttggaggtgtgttgcttgctcagatacaaattgcaattctgtattttatgtgataaaacatcctgacacccctgaaattttcttttccttttggggcaagtatatttggcattgattttctgcattttcaaggcttgaaatgaaatctgtgagcaggaagttcacagagtttatttcaagggataggtatatttctccagataaaattgaaagtgtgcagtgttattatataattgtgatgaagttgagctttgctagtttgaagtatctaataaagtttaacactttttaaaagatccttcaaaaagagcagttttcttgccatatgtaatattgttcagtgaatttttgaaatgaaatagatatttttcaaaaccacaaatgc
>chr22_KI270732v1_random:35,000-36,200                                                                                                                                                                                                                                                                                                                                                                                                                                                                                                                                                                                                                                caaatatacattgtcata      tataatgtttgtatatatgaaagt cctgtgggttatatatctgaaatcaaaattacaagtgttttgtaacttctataattaattttactctcaattttaagtaaacaagattagtttttgcacatgatgaatctctttattcagttaattactgccataattagaataccacttaaaacttgagaatattttcaagggtatttatgctaaacttacataagtgtgttgaaaagtccttttgtatatatgtagagattacatggtatgttaaattcatcaattttgcctaaatatcaagttatacttaagtagtgtccatacacaatttttttgcttgactgaactccaatatgtatattgggagtactttttaacattcataggttaaatggcctgttttggaaaatgatattaaaaatgtaattcagacgaatgcttagaataaagattaaagatgaatgagctttcatattaatcatcaatatgacaatcctaaagggaagcatgattttcaaatgtacctccttgtaagcaggataattcagaaatgcacagtgcatattagatatgggatttgagtcgtagtatatttctctaagaatgtaatttattgtactttcacatccaccccactgaatatgcagagtttaagatgcaatggccagaagacatgaaagtgtcactagaaggatgtggacgtgggctccgggactgttgatgatgactgtgatcttttggggtcatcaggggaatggacaaggccaaggtaagtgcaaggatgttctaattctttgagagttggatgcaaatttcactttatgatgaaattatgtgagtctttggtttgacgtttaagcaatttgttgtaatcttccgccaggtttttgttacagggtattgtaatgcctctgcaccagttaaattgaaatagagttaggtgtgagtaaatgcatcaaaattgaggccatctttccgaaattggcttttttctgttggaattcattatatatatatggaattctgttggaattcatatatatatatatatgcatatatatatgtatatatatatgaatatatatatgtatatatatatgaatatatatatgtatatatatatgaatatatatgtatatatatatgaatatatatatatgaatatatatacaccagatatgtatatc
>chr22:10,703,000-10,704,000                                                                                                                                                                                                                                                                                                                                                                                                                                                                                                                                                                                                                                                                                                                                                                                     tttttgcacatgatgaatctctttattcagttaattactgccataattagaataccacttaaaacttgagaatattttcaagggtatttatgctaaacttacataagtgtgttgaaaagtccttttgtatatatgtagagattacatggtatgttaaattcatcaattt gcctaaatatcaagttatacttaagtagtgtccatacacaatttttttgcttgactgaactccaatatgtatatggggagtactttttaacattcataggttaaatggcctgttttggaaaatgatagtaaaaatgtaattcagatgaatgctt_______agattaaagatgaatgagctttcatattaatcatcaatatgacaatcctaaagggaagcatgattttcaaatgtacctccttgtaagcaggataattcagaaatgcacagtgcatattagatatgggatttgagtcgtagtatatttctctaagaatgtaatttattgtactttcacatccaccccactgaatatgcagagtttaagatgcaatggccagaagacatgaaagtgtcactagaaggatgtggacgtgggctccgggactgttgatgatgactgtggtcttttggggtcatcaggggaatggacaaggccaaggtaagtgcaaggatgttctaattctttgagagttggatgcaaatttcactttatgatgaaattatgtgagtctttggtttgacgtttaagcaatttgttgtaatcttccgccaggtttttgttacaggatattgtaatgcctctgcaccagttaaattgaaatagagttaggtgtgagtaaatgcatcaaaattgaggccatctgtccgaaattggcttttttctgttggaattcattatatatatatggaattctgttggaattcatatatatatatatgaatatatatatgtacatatgtatatgaatatatatatgtacatatgtatatgaatatatatatgtacatat            gtata
                                                                                                                                                                                                                                                                                                                                                                                                                                                                                                                                                                                                                                                                                                                                                                                                                                                                                                                                                                                                                                 In the reverse sequence with the Chr20/22 primers, this base^      ^appears here because of the relative 7-bp deletion ^ here
                                                                                                                                                                                                                                                                                                                                                                                                                                                                                                                                                                                                                                                                                                                                                                                                                                                                                                                                                                                                                                                         At Position 1 in the bottom sequence        (Position 8) and this^      ^appears here, i.e. the g appears at Position 37 in the reverse sequence chromatogram instead of at Position 30.
